# An evolutionary perspective on anti-tumor immunity

**DOI:** 10.3389/fonc.2012.00202

**Published:** 2013-01-10

**Authors:** David J. Klinke

**Affiliations:** ^1^Department of Chemical Engineering, West Virginia UniversityMorgantown, WV, USA; ^2^Mary Babb Randolph Cancer Center, West Virginia UniversityMorgantown, WV, USA; ^3^Department of Microbiology, Immunology, and Cell Biology, West Virginia UniversityMorgantown, WV, USA

**Keywords:** proteomics, Bayesian inference, next generation sequencing, simulation

## Abstract

The challenges associated with demonstrating a durable response using molecular-targeted therapies in cancer has sparked a renewed interest in viewing cancer from an evolutionary perspective. Evolutionary processes have three common traits: heterogeneity, dynamics, and a selective fitness landscape. Mutagens randomly alter the genome of host cells creating a population of cells that contain different somatic mutations. This genomic rearrangement perturbs cellular homeostasis through changing how cells interact with their tissue microenvironment. To counterbalance the ability of mutated cells to outcompete for limited resources, control structures are encoded within the cell and within the organ system, such as innate and adaptive immunity, to restore cellular homeostasis. These control structures shape the selective fitness landscape and determine whether a cell that harbors particular somatic mutations is retained or eliminated from a cell population. While next-generation sequencing has revealed the complexity and heterogeneity of oncogenic transformation, understanding the dynamics of oncogenesis and how cancer cells alter the selective fitness landscape remain unclear. In this technology review, we will summarize how recent advances in technology have impacted our understanding of these three attributes of cancer as an evolutionary process. In particular, we will focus on how advances in genome sequencing have enabled quantifying cellular heterogeneity, advances in computational power have enabled explicit testing of postulated intra- and intercellular control structures against the available data using simulation, and advances in proteomics have enabled identifying novel mechanisms of cellular cross-talk that cancer cells use to alter the fitness landscape.

## INTRODUCTION

The transformation of a normal cell into a cancerous cell involves the acquisition of a series of genetic and epigenetic changes that daughter clones inherit (Hanahan and Weinberg, 2011). Next generation sequencing has reveal the breadth of genomic rearrangement that occurs in cancer (Stephens et al., 2009; Pleasance et al., 2010b; Gerlinger et al., 2012). These genetic and epigenetic changes can cause abnormal overexpression of proteins involved in cellular signaling pathways and can contribute to acquisition of these traits. Collectively, these genetic alterations rewire how cells interpret extracellular cues (Pawson and Warner, 2007; Klinke, 2010b) and subvert intracellular control mechanisms that are designed to maintain genetic integrity (Hollstein et al., 1991). It is thought that cells containing mutations in specific genes that impart an inherent proliferative advantage over cells of the host and that, over time, dominate a local cellular community. Demonstrating that a mutated gene, that is an oncogene, alters the replicative potential of a transformed cell supports this view (e.g., Muller et al., 1988; Gishizky et al., 1993). In order to inhibit the growth of malignant cells, drugs have been developed to promote cell death by targeting the oncogene in oncogene-addicted cells (Weinstein and Joe, 2008).

Demonstrating a durable clinical response in cancer using molecular-targeted therapies has been difficult. In patient groups stratified by a particular molecular biomarker, molecular-targeted therapies exhibit remarkable efficacy for a window of time in a subset of patients. For instance, overexpression of the epidermal growth factor receptor (EGFR) is observed in three-fourths of primary colorectal tumors (Hemming et al., 1992; Mayer et al., 1993) and provides support for targeting these cells using panitumumab, a monoclonal antibody against EGFR. The therapeutic window is short whereby almost all patients develop resistance within several months (Amado et al., 2008; Karapetis et al., 2008). *KRAS* (v-Ki-ras2 Kirsten rat sarcoma viral oncogene homolog) mutations are also a common occurrence in colorectal cancer. In a recent clinical study with panitumumab, 38% of patients that were initially negative for *KRAS* mutations developed circulating tumor cells that harbor detectable mutations in *KRAS* within 5–6 months (Diaz et al., 2012). A mathematical model was used to support the idea that resistance was due to drug-induced selection of cellular variants that harbored resistant mutations. A similar phenomena was observed in response to imatinib mesylate (Gleevec) in patients with chronic myeloid leukemia (Shah et al., 2002). While these are just two examples, the emergence of resistance to almost all molecular-targeted therapies in cancer brings a renewed interest in cancer as an evolutionary process (Merlo et al., 2006; Greaves and Maley, 2012).

Inherent in the view of cancer as an evolutionary process is that: (1) tumors consist of a heterogenous population of cells with different fitness for survival, (2) the competition among cells of a population is a dynamic process, and (3) there is a competitive landscape in the tumor microenvironment that select for variants with improved fitness. The fitness landscape includes competing for limited resources and intra- and extracellular mechanisms that are designed to maintain cellular homeostasis. While genetic sequencing technology has revealed the complexity and heterogeneity of oncogenic transformation, understanding the dynamics of oncogenesis and how cancer cells alter the selective fitness landscape remain unclear. In part, this uncertainty has been due to a scientific focus on how somatic mutations alter the inherent fitness of a cell to compete for limited resources and evade intracellular control structures (Nowak, 2006). Given the contemporary view of the degree of somatic mutations in cancer, acquiring oncogenes through random mutation also comes at a cost. Passenger mutations provide a rich source of neoantigens that can be recognized by the host immune system (Matsushita et al., 2012). Innate and adaptive immune cells comprise an extracellular control structure that is intended to restore cellular homeostasis within organ systems. Recent work suggests that malignant cells manipulate this control structure early in oncogenesis (O’Sullivan et al., 2012). In the following sections, we will describe how recent advances in technology have impacted our understanding of these three attributes of cancer as an evolutionary process. In particular, we will focus on how advances in genome sequencing have enabled improved quantification of cellular heterogeneity, how advances in computational power have enabled explicit testing of postulated intra- and extracellular control structures against the available data using simulation, and how advances in proteomics have enabled identifying novel mechanisms of cellular cross-talk that cancer cells use to alter the fitness landscape.

## A TUMOR CONTAINS A HETEROGENOUS POPULATION OF MALIGNANT CELLS

Cellular heterogeneity within tumors has been recognized for several decades (Fidler and Kripke, 1977). While early efforts focused on phenotypic and morphologic heterogeneity, improved experimental tools have expanded our contemporary understanding of non-genetic and genetic sources of cellular heterogeneity within a tumor. Non-genetic sources of cellular heterogeneity have been associated with sources of cellular stress within the tumor. The metabolic requirements for cell function coupled with the diffusion of nutrients and waste products within the tumor mass stratify the tumor into different regions: an actively proliferating outer shell, a senescent inner region, and a necrotic core (Venkatasubramanian et al., 2006). The conditions within the different regions impart one component of the selective fitness landscape. For instance, malignant cells have an improved ability fulfill energetic requirements under non-ideal conditions that include hypoxia, termed the Warburg effect (Warburg, 1956; Hsu and Sabatini, 2008). In addition, emerging evidence suggests that cellular stress associated with treatment promotes reversion of an epithelial to mesenchymal-like phenotype, a phenomenon associated with resistance (Knutson et al., 2006; Higgins et al., 2007; Ebos et al., 2009; Pàez-Ribes et al., 2009). Epithelial-to-mesenchymal transition (EMT) is a biological process involved in normal development. Elements of EMT are linked in cancer with the acquisition of stem cell properties, increased invasion, and metastasis (Mani et al., 2008). The acquisition of stem cell properties is also associated with a change in oncogene dependence, such as a loss in ErbB2 expression (Shipitsin et al., 2007) and a bypass of cellular dependence on ErbB1 signaling (Barr et al., 2008). This implies that clonally derived cells at different states of differentiation will vary in therapeutic sensitivity (Voulgari and Pintzas, 2009; Sharma et al., 2010). Taken together, these studies suggest that metabolic cross-talk between cells that compete for limited resources and alterations in cell phenotype due to EMT introduce a non-genetic source of variability in how cells contained within a tumor respond to therapy.

Genetic sources of heterogeneity among malignant cells arise from the action of mutagens, such as compounds found in tobacco and UV radiation. While different mutagens have different signatures of DNA damage (Greenman et al., 2007), the random nature of DNA damage and repair implies that there are multiple ways in which tumors can originate and that many cells within a population may harbor mutations, each with a different pattern of genetic alteration. To assess the diversity of cancers that arise in a particular organ, large collaborative efforts have focused on sequencing cancer genomes (e.g., Sjoblom et al., 2006; Ding et al., 2008; McLendon et al., 2008; Pleasance et al., 2010a). In early studies, resolution was limited to coding exons associated with protein-coding genes to identify base substitutions and small insertions or deletions (Sjoblom et al., 2006; Ding et al., 2008; McLendon et al., 2008). Next generation sequencing has enabled expanded genome coverage where chromosomal rearrangement and copy number changes could also be detected (Stephens et al., 2009; Pleasance et al., 2010a,b). While many of these studies still average over the collective tumor genome, the results highlight the heterogeneity among patients with a given cancer. In focusing on a specific cancer, a recent series of papers highlight the complexity of genomic rearrangement that occurs in breast cancer (Banerji et al., 2012;Curtis et al., 2012; Ellis et al., 2012; Shah et al., 2012; Stephens et al., 2012). Collectively the results suggest that the genomes of breast cancer cells are modified extensively such that individual breast cancers carry a few consistent and functionally characterized abnormalities and tens to thousands of other alterations about which little is known. More recently, the genomic alterations in single cells have also been reported, which highlight the heterogeneity among cells of a population (Gerlinger et al., 2012; Hou et al., 2012; Xu et al., 2012).

While these sequencing efforts have focused on clinically diagnosed tumors, autopsy studies suggest that alterations in the somatic genome may be much more prevalent within an organism than has been thought previously, a stage termed “occult cancer.” Nearly forty percent (39%) of women in their forties have histologic breast cancer and a similar percentage of men in their forties have histologic prostate cancer (Bissell and Hines, 2011). In support of occult cancer, these cancer sequencing studies highlight that many tumors emerge after a prolonged period of DNA damage and repair (Pleasance et al., 2010a). To illustrate the progressive change in the genome, phylogenetic trees associated with oncogenesis have been reconstructed using high resolution sequences (Greenman et al., 2012; Nik-Zainal et al., 2012). In breast cancer, the reconstructed phylogenetic trees suggests that a majority of the time associated with oncogenesis focuses on diversifying the tumor population and selecting among nascent cancer cells. The extent of genetic rearrangement in cancer cells also highlights the frequency of mutagen-induced DNA damage and repair. For instance in lung cancer, sequencing suggests that lung epithelial cells acquire an additional mutation for every 15 cigarettes smoked, despite intracellular mechanisms designed to restore the integrity of DNA (Pleasance et al., 2010b). As the pattern of mutations is not significantly different than expected by chance, the majority of these mutations are thought not to confer a selective advantage to the cancer cell. However, these passenger mutations may provide a source of potent tumor neoantigens, as was observed in carcinogen-induced mouse models of sarcoma (Prehn and Main, 1957; Matsushita et al., 2012). In addition, these sequencing studies also suggest that metastasis may occur at different stages in different cancers. Breast cancer metastasis may occur early in oncogenesis (Kuukasjarvi et al., 1997; Torres et al., 2007; Shah et al., 2009) while prostate cancer metastasis occurs late in oncogenesis (Liu et al., 2009). Clinically, cellular heterogeneity in cancer implies that clonally homogeneous tumors may respond more favorably to treatment using a molecular-targeted therapy while a clonally heterogeneous tumor increases the likelihood that the population contains tumor cells that can survive therapy-induced changes in the fitness landscape.

## THE TUMOR MICROENVIRONMENT IS A DYNAMIC SYSTEM

The second attribute of evolutionary processes is that the different cell types contained within the tumor microenvironment – stromal cells, malignant clones, and cells of the immune system – and their collective interactions create a dynamic system. This dynamic system interacts with a control structure associated with tissue homeostasis. Homeostasis is a central theme in physiology, where causal mechanisms are used to maintain the physiological state associated with life in the presence of external perturbations. These causal control mechanisms span multiple levels of organization (Klinke, 2010a) – from the cellular level, such as the intracellular mechanisms that control sodium and potassium concentrations in neurons following excitation, to the organisms level, such as organ-level mechanisms that regulate body temperature following changes in activity level. The challenge in tumor immunology is trying identify the immune-related control mechanisms that regulate the homeostatic composition of cells within an organ and how tumor cells interfere with this control structure.

To identify these control structures, one frequently creates a mental model of how one thinks a system behaves based upon prior knowledge of the system (i.e., a hypothesis); designs a controlled experiment; and acquires data to infer using statistics whether the mental model is a valid representation of the causal mechanisms that regulate system behavior. Conventionally, the mental models are “tested” against the observed data using tools of inferential statistics that were originally developed in the early 1900s (Neyman and Pearson, 1933; Fisher, 1935). Collectively, this process is called strong inference (Platt, 1964) or alternatively *in cerebello* model-based inference. There are five challenges with the conventional approach to identifying the control structure associated with tissue homeostasis and oncogenesis: (1) the interactions among cells occur locally in the tumor microenvironment, (2) robust control typically involves redundant mechanisms, (3) the control structures can be non-linear, (4) the roles that specific mechanisms play in regulating system response can change with time, and (5) many control structures are still unknown (i.e., lurking mechanisms exist). To address these challenges, we will first examine the weaknesses associated with the conventional *in cerebello* model-based inference and propose an alternative approach for inference that leverages contemporary advances in computational power.

One particular challenge in how classical tools of inferential statistics are used in practice is that one formulates the inference test in terms of two alternative hypotheses: the null hypothesis – the experimental perturbation introduces no change in the system – and an alternative hypothesis – the observed response is consistent with the proposed mechanistic hypothesis. If the data observed under control and perturbed conditions are sufficient different, the null hypothesis is rejected. Conventionally, the alternative hypothesis is then accepted. This conclusion depends on assuming that there are no other lurking mechanisms at work in the system. To highlight the problematic nature of this assumption, we consider recent controversial findings related to anti-tumor immunity. Two recent papers suggest that the adaptive immune system does not influence tumorigenesis and metastasis formation nor chemotherapy response in a spontaneous HER2-driven genetically engineered mouse model for breast cancer (Ciampricotti et al., 2011, 2012). These studies were in response to work that suggests that adaptive immunity does influence tumorigenesis (Shankaran et al., 2001; Dunn et al., 2002) and clinical response to chemotherapy (Apetoh et al., 2007; Obeid et al., 2007; Ghiringhelli et al., 2009; Mattarollo et al., 2011). de Visser and colleagues argue that transplantable models for cancer do not resemble established spontaneous tumors and use a genetically engineered mouse model (GEMM) where the mouse mammary tumor virus (MMTV) is used to induce tissue-specific expression of rat Her2 (Neu) in the mammary glands (i.e., the MMTV-NeuT model, Boggio et al., 1998). In contrast, Jacks and coworkers suggest that GEMMs of cancer may underestimate the mutational and antigenic load of most human cancers (DuPage et al., 2012).

Histological presentation of spontaneous breast cancer in the MMTV-NeuT may resemble the human equivalent (van Leeuwen and Nusse, 1995) but the molecular underpinnings of oncogenic transformation in humans may be completely different. While exome sequencing has yet to be reported, MMTV-NeuT tumors exhibit distinct and homogeneous patterns of gene expression that are unlike the human HER2+/ER-subtype (Herschkowitz et al., 2007). Oncogenes, like HER2, are a well-characterized subset of genes that upon amplification or silencing result in oncogenic transformation. While cancers commonly contain altered oncogenes, the random nature of DNA damage and repair implies that there is a mutational cost associated with malignancy. In thermodynamic terms, the conversion of one state to another state always comes at a cost, this cost is an increase in disorder (i.e., entropy)^1^. So while the MMTV promotes the expression of the oncogene, the available data suggests that the MMTV-NeuT GEMM of breast cancer does not reproduce the degree of mutational heterogeneity observed in human breast cancers. Moreover, HER2/Neu overexpression has been suggested to downregulate major histocompatibility complex (MHC) class I expression based upon clinical data (Maruyama et al., 2010), GEMMs (MMTV-Neu; Lollini et al., 1998), and cell models (Herrmann et al., 2004).

**FIGURE 1:**
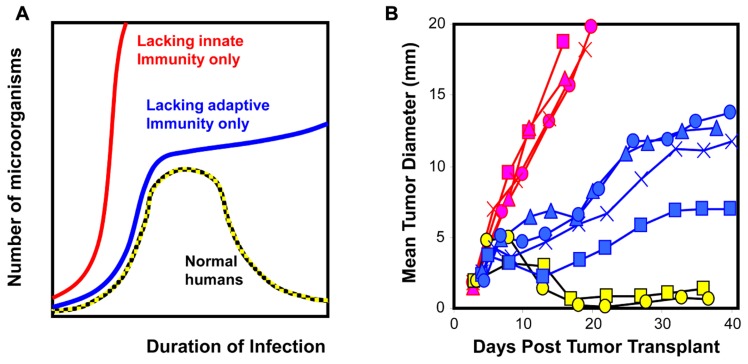
**Cellular homeostasis is a dynamic process that includes both innate and adaptive immunity**. **(A)** The dynamics associated with immune surveillance to microorganisms in humans and mice (Murphy et al., 2007). **(B)** Similarly, clones derived from 3’ methylcholanthrene-induced sarcomas exhibit different phenotypic dynamics upon transplantation [WT clones transplanted into RAG2^-/-^ hosts (red) and RAG2^-/-^ clones transplanted into WT hosts (blue and yellow; O’Sullivan et al., 2012)]. Restoring homeostasis that microorganisms or tumor cells are not present in the system requires both innate and adaptive immunity. The contribution of innate versus adaptive immunity changes with time; innate immunity dominates at early time points and initiates adaptive immunity that dominates at late time points. Results for highlighted clones imply that WT clones (red) have acquired ability to evade innate immunity and their ability to evade adaptive immunity is unknown. RAG2^-/-^ clones have acquired ability to evade adaptive immunity (blue) or are unable to evade innate and adaptive immunity (yellow).

To aid in interpreting the reported MMTV-NeuT GEMM data, we will consider a simple mathematical model for tumor growth. The fate of a malignant clone in a tissue microenvironment can be described as a dynamic system where competing cellular fates are regulated by a combination of intracellular mechanisms, such as initiation of cell proliferation or cell death, and extracellular control mechanisms, such as the role that immune cells play in eliminating microbes and foreign cells from the system. Mathematically, these causal mechanisms regulate the change in tumor size *(C_T_)* as a function of time:

$$\frac{d\,C_{T}}{d\,t}=\overset{\text{oncogenes alter k’s}}{\overset{︷}{\left(k_{p}-k_{d}\right)}}\cdot C_{T}-\underset{\text{innate immunity}}{\underset{︸}{k_{d\,I}\cdot C_{\text{II}}\cdot C_{T}}}-\underset{\text{adaptive immunity}}{\underset{︸}{k_{d\,A}\cdot C_{\text{AI}}\cdot C_{T}}},$$

where k_p_ and k_d_ are the propensity for a given transformed clone to either proliferate or die through an intrinsic mechanism within a period of time, respectively. The last two non-linear terms *k_dI_* · *C*_II_ · *C_T_* and *k_dA_* · *C*_AI_ · *C_T_* refer to the rates of cell death elicited by innate and adaptive immunity, respectively, and *C*_II_ and *C*_AI_ are the number of innate and adaptive immune cells within a given tissue volume. These non-linear terms are the product of three quantities: the abundance of immune cells within a given tissue volume, the abundance of cancer cells within a given tissue volume, and the propensity for a tumor cell to be killed following contact with an immune cell within a given period of time. In this simple model, the terms represent different biological control mechanisms. On the surface, innate and adaptive immunity may be considered redundant. However, as illustrated in **Figure 1**, the control exerted by innate and adaptive immunity changes with time. Our prior knowledge of relevant control mechanisms (i.e., that Neu overexpression downregulates MHC class I and the lack of diversity of neoantigens decreases the likelihood for an effective cytotoxic immune cell response) can be implemented in the model in the form of a reduced value for *k_dA_*. Then as the value of k_dA_ goes to zero, the presence or absence of adaptive immune cells does not alter the tumor growth trajectory. As these papers provide no information regarding the killing efficacy of cytotoxic T cell–tumor cell interaction, the data presented are insufficient to support the stated conclusions. As alluded to in this example, there are new methods for model-based inference that involve the use of mathematical models and simulation to test hypotheses.

In contrast to *in cerebello* model-based inference, *in silico* model-based inference is the statistical reasoning about our understanding of cause and effect in natural systems from experimental observation using computer simulation. Similar to a microscope that assists our natural ability to see small objects, mathematical models assist our natural intuition as they require an explicit statement of underlying assumptions and establish formal relationships between cause and effect. While mathematical modeling, *per se*, is not new to biology, there are recent advances in how our current understanding of a reactive system can be tested against the observed data. Conventionally called scientific hypothesis testing, this process aims to protect against the possibility that a discovery is based upon natural chance alone and not upon a new mechanism. The methods used for scientific hypothesis testing were developed in the early 1900s. These methods were well suited to the questions of the day, as we had very limited knowledge of biological systems and we were limited to pencil-and-paper calculations. Today, the intellectual landscape is different. High performance computing and high-throughput assays have fundamentally changed the way we study biology and motivate a contemporary approach. This contemporary approach is called *in silico* model-based inference and draws on ideas from high performance computing, statistics, and chemical kinetics. The combination of high performance computing with statistics is an active field of research that focuses primarily on data regression problems using correlative (or empirical) models (for a discussion of data regression in systems biology see Jaqaman and Danuser, 2006). Incorporating ideas drawn chemical kinetics enables *in silico* model-based inference and reshapes how these existing computational statistics tools are applied to problems of biological network inference.

In traditional chemical kinetic applications, mechanistic models of reaction networks are used for different objectives. Objectives include developing a mechanistically inspired empirical model for interpolating reaction data, developing reduced-order models of chemical kinetics to incorporate into more complicated models that account for fluid transport and reaction, and developing unbiased mechanistic models to aid in identifying key reaction steps that are at work under particular conditions. This last application is important if the resulting reaction model is going to be used to predict reactive behavior under new conditions and bears the most similarity to the challenges in biological network inference. It has also been known that mechanistically inspired empirical models have limited value in identifying novel reaction mechanisms as postulated mechanisms impose bias *a priori* (Green, 2007). This shortcoming of mechanistically inspired empirical models motivated generating mechanistic models of reaction networks using rule-based methods (Green, 2007). More recently, rule-based methods have also been embraced by the systems biology community (e.g., Faeder et al., 2009; Feret et al., 2009; Bachman and Sorger, 2011). One of the advantages of a rule-based method is that, instead of hand-crafting a reaction network using a set of implicit assumptions, computer algorithms are used to generate a reaction network given a set of reactants and a set of transformations that are thought to act within the system. It is relatively easy then to change the set of transformations and see how a different set of transformations impacts the predictive power of the resulting reaction network.

The rules represent fundamental transformations, such as protein–-protein interactions or elementary reactions steps, that are associated with the flow of chemical information within reaction networks. Each transformation has an associated rate constant that quantifies how quickly a transformation can occur given the presence of the reactants – a time scale. Moreover, the rate constants associated with each rule can be different. This implies that the overall flow of chemical information within reaction networks is governed by the slowest transformation. In traditional chemical kinetic applications, slow reactions are called rate-limiting steps. The rate-limiting steps correspond to sensitive levers within the reaction network that one can manipulate to achieve a desired objective – such as an improved conversion rate or selecting flow patterns within the reaction network to improve selectivity or yield of a desired product. Generally, this behavior is called the slaving principle [see comments on pg 6 of Klinke (2009, 2010a)].

In Klinke and Finley (2012), the time scales associated with the model parameters are linked to the fundamental transformations (i.e., protein–protein interactions or elementary reaction steps) that transmit chemical information within reaction networks. We show that only a subset of time scales can be uniquely identified using the observed data (i.e., exhibit two-sided bounded distributions). Transformations that are fast – such a pre-formed multi-protein complexes – and that are kinetically unimportant – such as extremely slow reactions – exhibit one-sided distributions. More importantly, this work demonstrates that the Adaptive Markov Chain Monte Carlo algorithm described in [Bibr B53] was the first to provide posterior distributions in the model parameters that are consistent with the slaving principle. Of note is that the prior statistical inference studies applied to biological network inference questions provide posterior distributions in the model parameters that have two-sided bounds all supposedly informed by data, such as a multivariate Gaussian distribution (e.g., Brown and Sethna, 2003; Brown et al., 2004; Gutenkunst et al., 2007; Vyshemirsky and Girolami, 2008; Toni et al., 2009; Toni and Stumpf, 2010; Calderhead and Girolami, 2011; Erguler and Stumpf, 2011). Given that none of the prior statistical inference studies provide “posterior” distributions that are consistent with the slaving principle, this raises the question as to whether these “posterior” distributions really reflect the data or whether they reflect an arbitrary selection of a prior or biased model formulation. For instance in Calderhead and Girolami (2011), the authors assume *a priori* that all of the postulated mechanistic steps encoded in the model are kinetically important – i.e., that there are no fast or extremely slow reactions. They also fixed *a priori* parameters that were structurally non-identifiable. Two-sided bounded distributions for all of the model parameters reported in these studies is not surprising as conventional Markov Chain Monte Carlo methods are used for regressing empirical models to data and tests of Markov chain convergence are applied to the model parameters.

As illustrated in Klinke et al. (2012), the *in silico* model-based inference approach can incorporate the best available domain knowledge, including competing hypotheses regarding topology, and search for all possible parameter combinations that provide model predictions consistent with the best available data. This paper illustrates three possible results from *in silico* model-based inference. First, the model predictions may be consistent with the observed data and only one competing topological hypothesis is favored, which suggests that the observed data is able to discriminate among the competing topological hypotheses and that the corresponding topology is of sufficient complexity to explain the observed data. The autocrine Tumor necrosis factor (TNF)-alpha feedback mechanism illustrates this result. Second, model predictions that are unable to match the observed data suggest that the topology is missing important connections, such as paracrine feedback mechanisms that may be important *in vivo* but have no effect under conventional *in vitro* conditions (e.g., see discussion of high density results at the top of pg 4). Third, the model predictions are consistent with the observed data but are unable to discriminate among competing topological hypotheses. The discovery of differential STAT1/STAT4 activation by interleukin (IL)-12 illustrates the third type of result. According to the editor of Science Signaling, this work “serves as an example of how mathematical modeling can refine our understanding of signaling pathways.” Ultimately, determining whether the topology of a reaction network can be uniquely identified from the available data is essential for identifying the right control structures at work in biological systems.

## THE SELECTIVE FITNESS LANDSCAPE IN CANCER CONTAINS INTRA- AND EXTRACELLULAR CONTROL ELEMENTS

The third attribute of evolutionary processes is that local cellular environment provides a selective fitness landscape for the retention or removal of malignant variants from a population. This local fitness landscape includes competing for limited resources – such as limited oxygen or glucose or stromal support – and active intra- and extracellular control mechanisms that aim to restore cellular homeostasis. Intracellular control mechanisms include p53, a protein that helps control genomic integrity and is mutated in more than half of all cancers (Hollstein et al., 1991), and the retinoblastoma tumor suppressor gene (pRb), which encodes a protein that regulates cell cycle (Friend et al., 1986). An example of an extracellular control mechanism is the role of innate and adaptive immunity in eliminating foreign or pathogenic organisms from the cellular population. As highlighted in an influential review (Hanahan and Weinberg, 2011), decades of cancer research have revealed how intracellular control mechanisms are evaded during oncogenesis. While it is well-known that tumor load limits the efficacy of immune cells in controlling tumor growth (e.g., Maccubbin et al., 1989; Pulaski and Ostrand-Rosenberg, 1998; van Elsas et al., 1999), our understanding of how cancer cells evade extracellular control mechanisms is still emerging.

**FIGURE 2:**
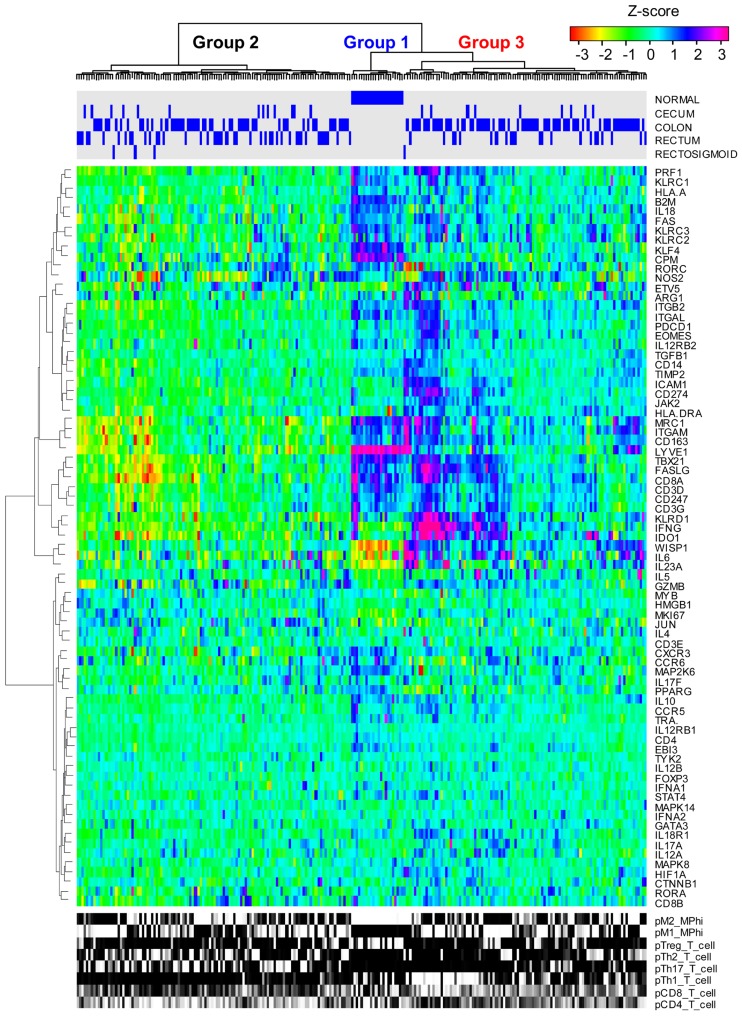
**Immune gene expression signatures in colorectal cancer clusters into three groups**. mRNA expression obtained from normal colorectal and cecum, colon, rectum, and rectosigmoid adenocarcinoma tissue samples (columns) were hierarchically clustered into three groups based upon the log2 median normalized expression ratio for genes (rows) related to cell-mediated cytotoxic immunity and tumor immunosuppression. The tissue of origin is highlighted by the blue bars on top and gene expression is shown as a row-normalized heatmap. Red denotes under-expressed and violet denotes overexpressed relative to the population mean. Dendrogram indicates the degree of similarity among genes (rows) or samples (columns) using the Ward’s minimum distance method in R.

**FIGURE 3:**
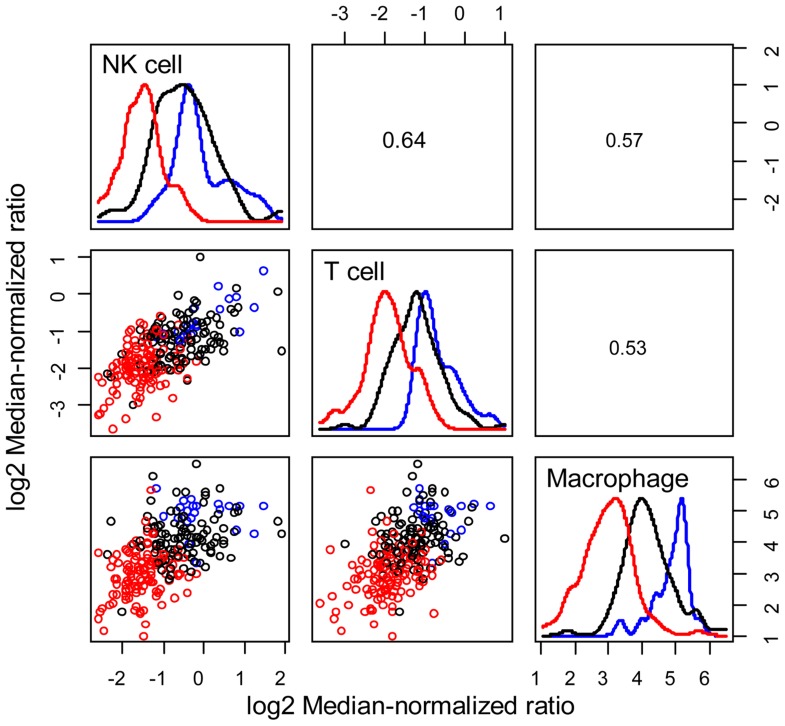
**Patient clusters exhibit different immune cell signatures**. Relative immune cell infiltrate was estimated based upon the average expression of genes associated with NK cells (*KLRD1*, *KLRC1*, *KLRC2*, *KLRC3*), T cells (*CD247*, *CD3G*, *CD3D*, *CD3E*), and macrophages (*CD14*, *CPM*, *MRC1*, *HLA-DRA*, *ITGAM*). Bivariate scatter plots are shown below the diagonal, marginalized histograms stratified by the three groups are shown on the diagonal, and correlation coefficients are shown above the diagonal. Results are colored by group (Group 1: blue, Group 2: black, Group 3: red).

**FIGURE 4:**
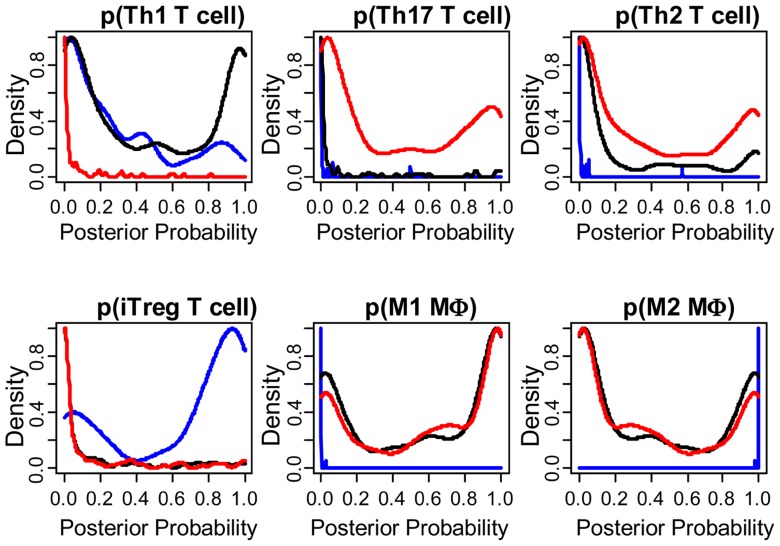
**The phenotype of immune cells within the tumor microenvironment are different among the three groups**. Posterior probability distribution for T helper cell and macrophage phenotypes stratified by group, where probability was based on mutually exclusive gene expression patterns that are associated with each cell subset. T helper cell subsets were based upon gene clusters associated with Th1 (*CD4, TBX21, EOMES, FASL, IFNG, IL10*), Th17 (*CD4, RORA, RORC, IL17A, IL17F*), Th2 (*CD4, GATA3, PPARG, IL4, IL5, IL6, IL10*), and iTreg (*CD4, FOXP3, RORC, TBX21, CCR6, IRF4, MYB, TGFB1, IL10, EBI3, IL12A*) differentiation (Wei et al., 2009). Macrophage subsets were based upon gene clusters associated with M1 (*IL6, IL12B, IL23A, NOS2, IDO1*) and M2 (*TIMP2, LYVE1, ARG1, KLF4, CD163*; Movahedi et al., 2010). Results are colored by group (Group 1: blue, Group 2: black, Group 3: red). Posterior probability for each patient is also shown in the bottom row of **Figure 2** (Gray scale where 0 = white and 1 = black).

As summarized in Eq. 1, immune-mediated tumor regression is proportional to the product of three terms: the number of tumor cells recognized by the host’s immune cells, the number of immune cells present in the tumor microenvironment that can elicit tumor-directed cytotoxicity, and the cellular efficiency of immune cells in eliciting tumor-directed cytotoxicity. Recent large-scale studies that aim to quantify the diversity of human cancer can also be used to identify the phenotype associated with different immune cells recruited to the tumor microenvironment. Understanding the composition and phenotype of cells contained within tumors may help inform future cancer immunotherapies (Kerkar and Restifo, 2012). As illustrated in **Figure 2**, mRNA expression results from 224 colorectal tumor and normal pairs reported as part of the Cancer Genome Atlas (TCGA) provide an overview of the immunological bias present in colorectal cancer (Muzny et al., 2012). These gene expression signatures can be used to infer the extent of natural killer (NK) cells, T cells, and tumor-associated macrophages recruitment into the tumor (see **Figure 3**) and the corresponding phenotype of immune cells within the tumor microenvironment (see **Figure 4**; Wei et al., 2009; Movahedi et al., 2010). Within this TCGA colorectal data set, three patient clusters were identified based upon a subset of genes associated with anti-tumor immunity and immunosuppressive mechanisms. Group 1 corresponds to normal tissue with a mixed Th1 and iTreg CD4+ T helper cell and M2 macrophage signatures. Groups 2 and 3 correspond to colorectal cancer samples with different immune signatures. Group 2 has a slightly lower gene signature associated with NK, T cell, and macrophage infiltrate compared to normal tissue samples while the immune cell infiltrate exhibits a preference for Th1 T helper cell and mixed M1 and M2 macrophage signatures. The gene signature associated with NK, T cell, and macrophage infiltrate is lowest in Group 3 and exhibits a mixed Th17 and Th2 T helper cell signature and a macrophage signature similar to group 2. Due to the short follow-up time associated with the colorectal study, the relationship between overall survival and these immune cell signatures is unclear. While these gene expression studies provide insight into the number and phenotype of immune cells present within the tumor microenvironment, identifying the control mechanisms that become altered during oncogenesis are difficult to identify from static snapshots of a biological state. Generally, identifying causal mechanisms at work in multi-component systems is one of the most pervasive problems in the analysis of physiological systems (Khoo, 2000).

In engineering, this problem is called a system identification problem where causal relationships between system components are inferred from a set of input (i.e., biological cue) and output (i.e., response) measurements (Khoo, 2000). In context of anti-tumor immunity, an input may be the influx of cytotoxic T lymphocytes that recognize tumor-specific antigens and an output may be tumor regression. Many approaches exist for the identification of open-loop systems, where a change in input causes a unique change in output. Reductionist methods have revealed a wealth of knowledge regarding how isolated components of physiological systems respond to biological cues. However, the different cell types contained within the tumor microenvironment constitute a closed-loop system, as implied by the observation that tumor load influences the efficacy of immune cells that enter the tumor microenvironment. A closed-loop system is defined as a multi-component system where the output (i.e., response) of one component provides the input (i.e., biological cue) to another component. Closed-loop systems are particularly challenging as it is impossible to identify the relationships among components of a system based upon overall input (e.g., T cell infiltrate) and output (e.g., tumor regression) measurements. One of the reasons for this is that changes in the internal state of the system, such as an increase in biological cues associated with tumor load, may alter the response of the system to a defined input, such that there is not a direct causal relationship between overall system input and output. Historically, the causal mechanisms underlying the behavior of closed-loop systems in physiology have been identified via ingenious methods for isolating components within the integrated system, that is methods for “opening the loop.” A classic example of this is the discovery of insulin by Dr. Frederick Banting and Charles Best in 1921 and it’s role in connecting food intake to substrate metabolism (Roth et al., 2012). In this case, the biological cues – insulin and glucagon – that facilitate communication between components – endocrine pancreas, liver, and muscle – can be easily assayed in the blood. Observing and regulating these endocrine hormones in the blood enable one to disassemble the closed-loop system into a series of coupled open-loop systems. Each open-loop system responds in defined ways to biological cues, as depicted in the minimal model for the regulation of blood glucose (Bergman et al., 1979).

There are two key differences in applying systems identification methods to help identify the control mechanisms that regulate anti-tumor immunity compared with the control mechanisms that regulate substrate storage and metabolism. First, the biological scales are different: coordinated substrate storage and metabolism is an organ-level phenomenon while anti-tumor immunity is a cell population-level phenomenon. Second, the cross-talk among components occurs locally through secreted proteins or cell-to-cell contact. While the dynamics of cell-to-cell interactions within the tumor microenvironment can be observed using intravital live imaging (Engelhardt et al., 2012), the biochemical cues responsible for cell cross-talk are more difficult to identify *in vivo*. Moreover, samples from the peripheral blood may not be representative of the local biological cues responsible for cell cross-talk. Conventionally, immunohistochemical methods have been used to identify local biological cues present in the tissue microenvironment, a discovery process associated with experimental bias. The experimental bias stems from the fact that the method for detecting a local biological cue must be selected *a priori* and that methods for detecting this biological cue must exist (i.e., an antibody must exist). Similar to the development of rule-based modeling methods as a way to minimize bias, proteomics provide less biased methods for identifying local signaling mechanisms that contribute to homeostatic control.

Proteomic methods have been incorporated into a variety of workflows for identifying biochemical cues that underpin cell population-level control mechanisms. Analogous to immunohistology, recent work describes imaging protein, lipid, and small molecule profiles in biological tissues using direct laser-assisted ionization followed by time-of-flight mass spectrometry (Nemes et al., 2010; Stauber et al., 2010). The distribution of lipid and small molecular profiles can be obtained at a lateral resolution of 350–35 μm (Campbell et al., 2012). However, discriminating between extracellular and intracellular localization and identifying higher molecular weight proteins is difficult given the current technology, although improvements are likely (Jungmann and Heeren, 2012). Another approach is to create minimal co-culture model systems that reproduce critical aspects of the cellular cross-talk that occurs within the tumor microenvironment. To identify mechanism of resistance to anti-cancer therapies, Golub and coworkers assayed the *in vitro* response of 45 different cancer cell to 35 anti-cancer drugs while co-cultured with one of 23 different stromal cell lines (Straussman et al., 2012). They used a reverse phase protein array to identify that stromal cells secrete hepatocyte growth factor (HGF) that confers tumor cell resistance to RAF inhibitors (e.g., vemurafenib). This mechanism for cellular cross-talk was supported by immunohistology results showing that stromal cell expression of HGF correlates with innate resistance to RAF inhibitor treatment in human melanoma. While the results highlight that local paracrine cues can influence therapeutic response, using a reverse phase protein array still assumes that the proteins responsible for the observed behavior are measured by the array. As a less biased alternative, mass spectrometry can be used to identify proteins that are secreted within the co-culture system. In Kulkarni et al. (2012), Klinke and coworkers used a 2D-gel electrophoresis MALDI-TOF/MS workflow in conjunction with a high content co-culture assay to identify that malignant melanocytes secrete exosomes and Wnt-inducible signaling protein-1 (WISP1). Exosomes are nanometer-sized endogenous membrane vesicles that are produced by a diverse range of living cells and are thought to play key roles in shaping intercellular communication, such as immunity (Théry et al., 2009). By co-culturing the malignant melanocytes with a Th1 cell line, they found that WISP1 inhibits the functional response of the Th1 cell to IL-12. From a systems identification perspective, *in silico* model-based inference was used to confirm that, in isolation, the Th1 cell line can be described as an open-loop system and that the *in vitro* co-culture model recreates a closed-loop system. *In silico* model-based inference was also used to infer that WISP1 is expressed at the periphery of B16-derived tumors *in vivo*, a similar pattern of WISP1 expression was observed in human melanoma. In addition to secreting WISP1, they also found that the B16 model for melanoma overexpresses one component of the IL-12 receptor, IL12Rβ2, that creates a local cytokine sink for IL-12. In other work, they report that STAT4 is phosphorylated irreversibly, creating a short term memory to IL-12 signaling (Klinke et al., 2012). The duration of this memory is limited by cell proliferation. Other groups have shown that local delivery of IL-12 to the tumor microenvironment promotes tumor regression in the B16 melanoma model (Kerkar et al., 2011, 2010) and in the El4 thymoma model (Pegram et al., 2012). Collectively, these studies imply that signaling by endogenous IL-12 within the tumor microenvironment may help maintain T cell polarization when cognate tumor antigens induce T cell proliferation (Wang et al., 2007) and that manipulating this extracellular control mechanism may impart a survival advantage to the collective tumor population. In summary, these examples illustrate that coupling co-culture models with proteomics can uncover important local control mechanisms and that choosing a particular proteomics workflow involves a trade-off between selecting the degree of abstraction from reality in designing the experimental system and observing biochemical cues, given the current limits of the technology.

## CONCLUSION

It has been over a decade since molecular-targeted therapies revolutionized the treatment of cancer. The clinical reality observed in intervening years has dampened the initial enthusiasm, as efficacy is limited to defined patient groups and durable response is difficult to achieve. Contemporary understanding of oncogenesis paints a more complex picture of cancer as an evolutionary process. As an evolutionary process, cancer has three hallmark characteristics: (1) that malignant cells within the tumor microenvironment are heterogeneous, (2) that interactions among cells within the tumor microenvironment comprise a dynamic system, and (3) that intra- and extracellular control mechanisms constitute a selective fitness landscape that determines the survival of cells within the tumor microenvironment. Innate and adaptive immunity function as important extracellular control mechanisms. Observed in a subset of melanoma patients, durable response to a new immunotherapy provides hope that restoring these extracellular control mechanisms can be used as an effective weapon in the battle against cancer (Hodi et al., 2010). However, increasing the subset of patients that receive clinical benefit requires an improved understanding of cancer as an evolutionary process. Here, we have reviewed some of the emerging technologies that have improved our understanding of these evolutionary hallmarks. A common theme in this review is how new technology improves our ability to limit unintended bias. At the same time, advances in computing power motivate new methods for model-based inference that leverage the rich body of knowledge accumulated over decades of oncology and immunology research. Only through an integrated approach, will we be able to deliver a true revolution in cancer treatment.

## Conflict of Interest Statement

The author declares that the research was conducted in the absence of any commercial or financial relationships that could be construed as a potential conflict of interest.

## Footnotes

1 To make the thermodynamic analogy, we assume that the genome is a closed system and initially is comprised of a single genetic microstate. Mutations are introduced through a random process associated with DNA damage and repair. The acquisition of a genetic microstate that exhibits an improved fitness using this random process is also associated with the population acquiring additional microstates that exhibit neutral or negative fitness. Entropy is proportional to the number of possible genetic microstates that cells within a population can occupy. The analogy implies that adaptive immunity is an entropy detector. Cancers that exhibit a simple mutation signature may not engage adaptive anti-tumor immunity but may be more responsive to molecular-targeted therapy. Alternatively, cancers that exhibit a complicated mutation signature may not exhibit a durable response to molecular-targeted therapy and may be controlled by re-establishing adaptive anti-tumor immunity.
